# Children, Acid and Alkaline

**Published:** 1900-05

**Authors:** 


CHILDREN, ACID AND ALKALINE. "Health, the Golden Mean." The Law of Diet Selection, Contraria. The Therapeutic Law, Semilia. By THOMAS C. DUNCAN, M. D., Ph.D., LL.D., Professor of Medicine and Diseases of the Chest, Dunham Medical College; Consulting Physician to Chicago Foundling's Home, St. Anthony's Hospital, Cook County Hospital, etc., etc. Philadelphia: Boericke & Tafel. 1900.

This book, bearing the singular title, Children, Acid and Alkaline, contains its germ of truth—not a new truth, however, and so obscured by fantastic theory that one is amused rather than instructed in perusing it. Permit the reviewer to call the attention of Buffalo's zealous health commissioner to this quotation from page 41, on the feeding of alkaline children: "The long tube with a hard nipple should be selected." Have the children of Buffalo been properly classified? Possibly the prohibition of the sale of the long tube nursing-bottle is so drastic a measure, and there may be infants in this city whose physical welfare is threatened by such a sweeping interdiction. Might not the ordinance be so modified, that if a woman could prove that she had an alkaline child, a long tube nursing-bottle would be permitted? M. J. F.

